# Effects of Compression Garments on Balance Control in Young Healthy Active Subjects: A Hierarchical Cluster Analysis

**DOI:** 10.3389/fnhum.2020.582514

**Published:** 2020-11-12

**Authors:** Kévin Baige, Frédéric Noé, Noëlle Bru, Thierry Paillard

**Affiliations:** ^1^Laboratoire Mouvement, Equilibre, Performance et Santé (EA 4445), Université de Pau et des Pays de l’Adour/E2S UPPA, Tarbes, France; ^2^Laboratoire de Mathématiques et de leurs Applications, UMR CNRS 5142, Université de Pau et des Pays de l’Adour/E2S UPPA, Pau, France

**Keywords:** sports, posture, postural balance, sensorimotor control, sensory reweighting

## Abstract

There is controversy about the influence of compression garments on balance control. A positive influence was reported in elderly and injured individuals, whereas no beneficial effects were observed in young healthy active subjects, which is likely due to the large inter-individual differences in these subjects. Hence, this study investigated the acute effects of compression garments on balance control in young healthy active subjects by addressing the issue of heterogeneity of individuals’ responses to the wearing of compression garments. Thirteen young, healthy, active subjects were recruited. They stood on a force plate which recorded the center of foot pressure displacements in a monopedal stance with the eyes closed and on a wobble board with the eyes open, while wearing compression garments or not. Statistics were first calculated with the data from the whole sample. A hierarchical cluster analysis was also performed in order to categorize the participants’ behaviors into subgroups with similar characteristics. The whole group analysis showed that there were no significant effects attributed to compression garments. The clustering analysis identified distinct and homogeneous subgroups of participants. Only participants who swayed the more at baseline benefited from the wearing of compression garments to improve their balance control. These participants might have either a gravity-dependent preferred sensorimotor strategy with an exploratory postural behavior or poorer balance/proprioceptive abilities. Since poor balance control is a predictor of sports injury risk, wearing compression garments during sports practice could be viewed as a potential prevention strategy for individuals at risk.

## Introduction

The somatosensory system highly contributes to balance control. It provides information about body movements that lead to small-sized tissue deformation and activation of cutaneous, muscle, and articular mechanoreceptors (Krishnamoorthy et al., 2002). From an ecological perspective (Gibson, 1966), the somatosensory system should not be reduced to the mere consideration of nerve cells (i.e., mechanoreceptors): it should be considered at a more expansive scale while integrating nerve, muscle, and connective tissue cells in a complex tensegrity assemblage (Palatinus et al., 2014; Turvey and Fonseca, 2014). Connective tissue is of particular importance since it has a uniting function by binding every cell to its neighbors in order to form a whole medium that enables the transmission and the distribution of mechanical forces within the tensegrity assemblage (Palatinus et al., 2014; Turvey and Fonseca, 2014). Such a unification gives emphasis of relations between individual cells, which facilitate the pickup of information about body motion that emerges from interactions between perception, action, information, and task goal (Riley et al., 1999). In this context, the wearing of compression garments may change the tension–compression relations in the tensegrity assemblage. Actually, the overall constriction provided by compression garments acts as a mechanically supportive framework sensitive to body movements that can activate interacting cutaneous mechanoreceptors that individually would not have been activated. Hence, compression garments can enhance the perception of somatosensory information and influence balance control positively by reducing body sway during quiet standing (Kuster et al., 1999; Michael et al., 2014; Woo et al., 2017, 2018).

These beneficial effects of compression garments on balance control would be most pronounced among elderly and injured subjects (Kuster et al., 1999; You et al., 2004; Palm et al., 2012; Woo et al., 2018) and high-level athletes (Michael et al., 2014). These results suggest that only subjects with either sensory deficits, such as elderly and injured subjects, or exceptional sensory acuity and/or sensory reweighting mechanisms, such as high-level athletes (Paillard, 2017, 2019), would be able to benefit from compression garments to improve balance control. By comparison, “ordinary” active subjects such as young, healthy, non-injured subjects and non-elite/recreational athletes would not benefit from compression garments to improve balance control (Hijmans et al., 2009; Cavanaugh et al., 2016; Jaakkola et al., 2017).

Further studies about the influence of external devices that stimulate cutaneous receptors have shown that there was a great variability between individuals’ responses to cutaneous stimulation (Rogers et al., 2001; You et al., 2004; Pavailler et al., 2016). Hence, one can assume that the effects of compression garments on balance control would be subject-dependent in active subjects. This could explain the absence of a significant effect as reported by studies about the influence of compression garments on balance control in active subjects (Hijmans et al., 2009; Cavanaugh et al., 2016; Jaakkola et al., 2017). Actually, these studies were performed with standard group ensemble average statistical approaches that do not allow to specifically address the issue of heterogeneity of individuals’ postural behavior to the wearing of compression garments.

The purpose of this study was therefore to investigate the effects of compression garments on balance control in young, healthy, active subjects by addressing the issue of inter-individual variability in the ability to take advantage of compression garments to improve balance control. To this end, a hierarchical cluster analysis was performed. The clustering analysis is a multivariate statistical method which categorizes the participants’ behaviors into subgroups with similar characteristics, thus facilitating the investigation of differences in individual responses by enabling the identification of natural groupings that may exist in a whole sample (Watelain et al., 2000; White and McNair, 2002). It was hypothesized that the wearing of compression garments differently affected balance control in active subjects.

## Materials and Methods

### Participants

Thirteen male young, healthy, active subjects having recreational handball practice (age: 27.9 ± 8.36 years old, height: 178.6 ± 7.89 cm; body mass: 79.62 ± 14.28 kg; training hour per week: 3 h; mean ± SD) participated voluntarily in the experiment. The exclusion criteria included any neuromuscular impairments and/or ankle, knee, and hip trauma in the past 2 years and medication that might influence balance. The participants were also asked to avoid strenuous activity and the ingestion of alcohol or/and excitatory substances 24 h before the experimental session. All the participants voluntarily signed an informed consent before starting the experiment, which was in accordance with the Declaration of Helsinki. All procedures were approved by and performed in accordance with the relevant guidelines and regulations of the University of Pau and Adour Countries Ethics Committee.

### Apparatus and Procedure

The participants were asked to sway and move as little as possible when standing barefoot in a monopedal stance on their non-dominant leg (i.e., the supporting leg, which was determined as the leg which is not used to kick a ball) for 25 s on a force platform (Stabilotest^®^ Techno Concept^TM^, Mane, France) which sampled the center of foot pressure displacements at 40 Hz. Two postural tasks were conducted in a counterbalanced order: a stable task where the participants stood on stable ground with the eyes closed (while keeping their gaze straight ahead) and an unstable task where they stood on a wobble board with a diameter of 40 cm and a height of 8 cm (Balance-board, Sissel^®^ GmbH, Bad Dürkheim, Germany) with the eyes open (while looking at a fixed-level target at a distance of 2 m). These postural tasks were chosen because tactile cues provided by a light touch or by garments that stimulate cutaneous receptors are more effective in conditions of increased sway, i.e., when standing on an unstable surface and/or when a sensory pathway critical for standing posture is perturbed (e.g., when vision is occluded) (Rogers et al., 2001; Michael et al., 2014; Woo et al., 2018). For accurate and similar feet positioning between all subjects, the foot was placed according to precise landmarks on the force platform and the wobble board. All postural tasks were performed with or without wearing compression garments (COMP and REF condition respectively). In the COMP condition, calf compression sleeves (Booster, BV sport^®^, Saint Etienne, France) made of 69% polyamide, 21% elastane, and 10% yarn were worn by the participants. The pressure was 0 mmHg at the ankle, 20 mmHg at the external spinning, and 25 mmHg at the internal spinning of the calf. In order to avoid any learning effect, the participants had to perform a familiarization session before data acquisition while performing two trials in each postural task (Cug and Wikstrom, 2014). Center of pressure (COP) surface area (S: 90% confidence ellipse), mean center of pressure velocity along the medio-lateral (VX), and antero-posterior (VY) axes were calculated as linear measures characterizing balance control and postural sway; within the framework of a quiet stance paradigm, the lower these parameters, the lower amount of postural sway and the more efficient the balance control (Paillard and Noé, 2015). The analysis of the dynamical features of postural sway variations was calculated from the sample entropy (SampEn). Sample entropy was calculated from the resultant COP velocity using the method proposed by Ramdani et al. (2009), with *m* = 3 and *r* = 0.2. SampEn provides information about the regulation processes involved in balance control and attention investment. Low SampEn values characterize repetitive patterns of sway movements and reflect a strong cognitive contribution to balance control with reduced adaptive abilities. High SampEn values are indicative of a more automatic and adaptive control of balance (Ramdani et al., 2009; Vaillancourt and Newell, 2002). The relative difference of each center of pressure parameter between both conditions [relative difference = 100 × (COMP – REF)/REF] was also calculated. Relative difference is an easily interpretable descriptor, which limits the influence of the heterogeneity between participants in the REF condition and makes it easy to differentiate participants who benefit from CG wearing (negative value of relative difference of linear center of pressure metrics and positive value of relative difference of SampEn) and those who do not (positive value of relative difference of linear center of pressure metrics and negative value of relative difference of SampEn).

### Statistical Analysis

Data were analyzed in two steps. The first step consisted of analyzing the influence of experimental conditions (REF and COMP) on dependent variables (S, VX, VY and SampEn) with a standard group ensemble average method (whole group analysis). Normality was tested using Shapiro–Wilk test. As the dependent variables did not meet the assumption of normal distribution, non-parametric Wilcoxon signed-rank tests were applied to determine differences between the REF and the COMP conditions in terms of the relevant dependent variable.

A hierarchical cluster analysis was performed in a second step to classify the participants’ behavior into subgroups with similar characteristics (Watelain et al., 2000; White and McNair, 2002). A normalized principal component analysis was first used to reduce the dimensionality of the data prior to performing the hierarchical cluster analysis based on the five first principal component scores. COP parameters in the REF condition (S, VX, VY, and SampEn) and the relative differences (RD) of each COP parameter (RD_S, RD_VX, RD_VY, and RD_SampEn) between both conditions were used to produce a 13 × 8 input matrix for the PCA [13 (number of participants) × 8 (input variables)]. Once the number of clusters was identified, univariate tests were performed on dependent variables to determine which parameters best determine the cluster placement due to a significant difference from whole group mean (Lê et al., 2008). The stable and the unstable postural tasks were analyzed independently as two separated datasets. Statistical analyses were performed with R statistical software using FactoMineR Package (Lê et al., 2008). The significance level was set at *p* < 0.05.

## Results

Table 1 illustrates the mean values of the center of pressure parameters in the whole sample with the characteristics of each cluster. When inferential statistics were conducted on the whole group, no significant differences were observed between REF and COMP conditions in both the stable and the unstable postural tasks.

**TABLE 1:** Center of pressure (COP) parameters of the whole group with cluster characteristics in the different postural tasks and experimental conditions.

			***n***	**REF**	**COMP**	**RD (%)**
Stable task	S (mm^2^)	Whole group	13	2,007.48 (1,120.31)	1,865.7 (801.08)	8.04 (43.41)
		Cluster 1	10	1,506.21 (580.01)^a^	1,807.212 (815.42)	22.67 (35.66)^a^
		Cluster 2	3	3,678.40 (756.58)^a^	2,060.50 (885.60)	−40.75 (30.72)^a^
	VX (mm s^–1^)	Whole group	13	65.07 (16.61)	64.55 (18.07)	−0.13 (14.54
		Cluster 1	10	57.96 (10.25)^a^	58.3 (12.87)	1.11 (14.73)
		Cluster 2	3	88.76 (9.32)^a^	85.38 (19.17)	−4.28 (16.09)
	VY (mm s^–1^)	Whole group	13	52.51 (18.5)	51.73 (16.99)	3.70 (32.27)
		Cluster 1	10	43.52 (7.58)^a^	49.74 (17.77)	13.71 (29.33)^a^
		Cluster 2	3	82.47 (6.51)^a^	58.37 (14.99)	−29.68 (14.37)^a^
	SampEn	Whole group	13	1.26 (0.18)	1.26 (0.22)	0.37 (12.04)
		Cluster 1	10	1.24 (0.19)	1.23 (0.23	−0.56 (13.73)
		Cluster 2	3	1.31 (0.20)	1.35 (0.16)	4,00 (4.81)
Unstable task	S (mm^2^)	Whole group	13	700.55 (384.28)	693.70 (266.23)	6.96 (29.87)
		Cluster 1	4	619.50 (101.37)	538.83 (95.28)	−13.02 (8.03)^a^
		Cluster 2	3	679.70 (344.44)	874.23 (375.62)	32.30 (24.16)
		Cluster 3	5	558.74 (209.10)	608.32 (118.46)	15.77 (30.88)
	VX (mm s^–1^)	Whole group	13	41.04 (11.70)	39.15 (8.53)	−0.75 (23.71)
		Cluster 1	4	47.38 (6.43)^a^	38.53 (3.87)	−18.24 (5.97)^a^
		Cluster 2	3	34.95 (8.72)	44.79 (14.95)	26.60 (13.41)^a^
		Cluster 3	5	33.90 (2.15)	34.94 (6.17)	3.54 (19.96)
	VY (mm s^–1^)	Whole group	13	26.83 (8.49)	26.34 (5.68)	1.01 (12.73)
		Cluster 1	4	28.93 (4.39)	26.63 (2.6)	−7.14 (10.50)^a^
		Cluster 2	3	29.40 (5.48)	30.13 (4.19)	3.16 (5.76)
		Cluster 3	5	19.46 (1.70)^a^	21.51 (2.97)	10.42 (9.86)
	SampEn	Whole group	13	1.34 (0.22)	1.39 (0.18)	4.71 (9.93)
		Cluster 1	4	1.34 (0.10)	1.37 (0.17)	2.52 (9.3)
		Cluster 2	3	1.59 (0.24)^a^	1.56 (0.15)	−1.04 (10.07)
		Cluster 3	5	1.17 (0.14)^a^	1.30 (0.19)	11.01 (9.62)

*Data are expressed as mean (SD). S, COP surface area; VX and VY, mean COP velocity along the medio-lateral and antero-posterior axes, respectively; SampEn, sample entropy; *n*, frequencies for the whole group and subgroups; REF, reference condition; COMP, compression garments condition; RD, relative difference between both conditions [RD = 100 × (COMP – REF)/REF]. ^a^Variable identified as determinant of cluster placement due to a significant difference from the whole group mean (*p* < 0.05).*

**FIGURE 1:**
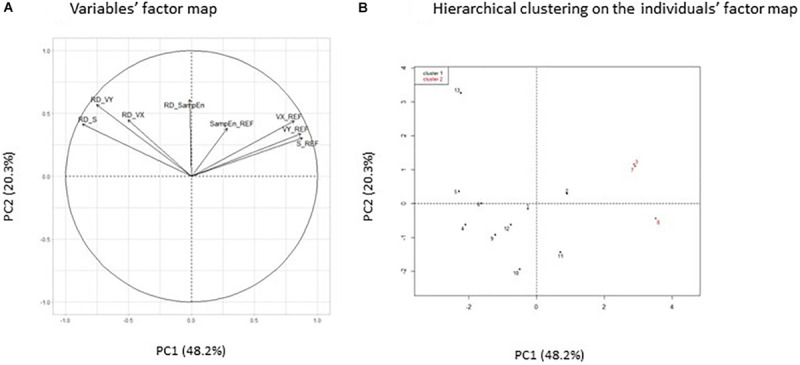
Variables’ factor map **(A)** of the principal component analysis applied on center of pressure (COP) parameters in the stable postural task and hierarchical clustering on the factor map **(B)**. Individuals from clusters 1 and 2 are represented by black and red dots, respectively. S_REF, COP surface area in the REF condition; VX_REF, mean COP velocity along the medio-lateral axis in the REF condition; VY_REF, mean COP velocity along the antero-posterior axis in the REF condition; RD_S, RD_VX, RD_VY, and RD_SampEn, relative difference between both conditions [RD = 100 × (COMP – REF)/REF] of S, VX, VY, and SampEn variables.

In the stable postural task, the principal component analysis resulted in two components that explained 68.5% of the total variance of the original dataset. The first principal component accounted for 48.2% of the total variance. Among the variables that loaded this component, S_REF, VX_REF, and VY_REF were positively correlated, whereas RD_S and RD_VY were negatively correlated (Figure 1A). The second principal component explained 20.3% of the total variance and was mainly loaded with two variables, RD_VY and RD_SampEn, that were positively correlated. Figure 1B illustrates the individuals’ factor map with the results from the hierarchical cluster analysis. Two clusters were identified with a heterogeneous distribution of individuals among the clusters (cluster 1, *n* = 10; cluster 2, *n* = 3). The center of pressure parameters that best determined group placement was acting on the first principal component axis (S_REF, VX_REF, VY_REF, RD_S, and RD_VY). Individuals from cluster 1 had low values of linear center of pressure metrics in the REF condition (S_REF, VX_REF, and VY_REF) and positive values of relative difference between both conditions (RD_S and RD_VY). Individuals from cluster 2 had high values of linear center of pressure metrics in the REF condition and negative values of relative difference.

**FIGURE 2:**
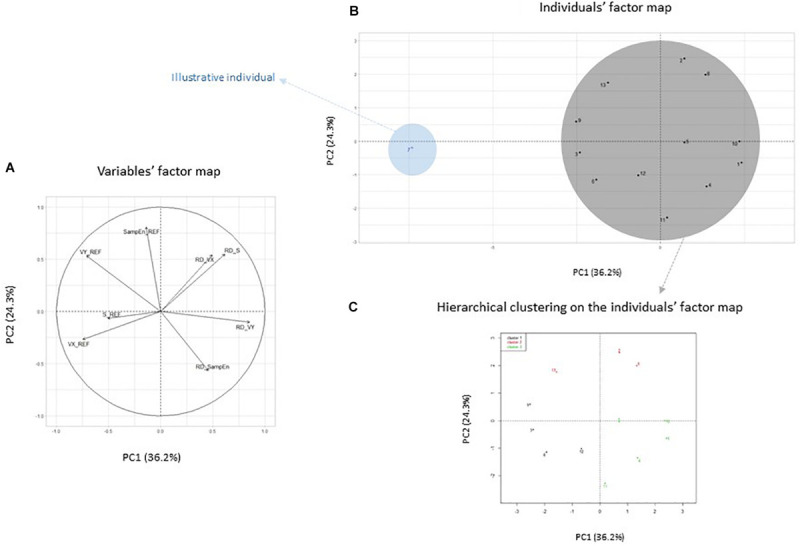
Variables’ factor map and individuals’ factor map **(A,B)** of the principal component analysis applied on center of pressure (COP) parameters in the unstable postural task and hierarchical clustering on the factor map **(C)**. Individuals from clusters 1 and 2 are represented by black and red dots, respectively. S_REF, COP surface area in the REF condition; VX_REF, mean COP velocity along the medio-lateral axis in the REF condition; VY_REF: mean COP velocity along the antero-posterior axis in the REF condition; SampEn_REF; RD_S, RD_VX, RD_VY, and RD_SampEn, relative difference between both conditions [COMP = 100 × (CG – REF)/REF] of S, VX, VY, and SampEn variables.

In the unstable postural task, we left out participant number 7 from the analysis. As illustrated in Figure 2B, he was identified as an outlier on several dependent variables of the initial dataset (VY_REF, S_REF), which influenced the construction of the space too significantly. Participant number 7 was entered as an illustrative individual, and 12 active individuals were included in the principal component analysis and cluster analyses. The principal component analysis resulted in two components that explained 60.6% of the total variance of the original dataset. The first principal component accounted for 36.2% of the total variance and was mainly loaded with four variables: RD_S and RD_VY that were positively correlated and VX_REF and VY_REF which were acting in the opposite direction. The second principal component explained 24.3% of the total variance and was mainly loaded with five variables: RD_S, RD_VX, VY_REF, and SampEn REF that were positively correlated and RD_SampEn which was acting in the opposite direction (Figure 2A). Three clusters were identified with a fairly homogeneous distribution of individuals among the clusters (cluster 1, *n* = 4; cluster 2, *n* = 3, cluster 3, *n* = 5). The COP parameters that best determined group placement were acting on the PC1 axis (S_REF, VX_REF VY_REF, RD_S, and RD_VY). Individuals from cluster 1 differed from the whole group by having higher values of VX_REF and lower values of RD_VX and RD_VY. Individuals from cluster 2 had higher values of SampEn_REF and RD_VX than those of the whole group. Finally, individuals from cluster 3 had lower values of SampEn_REF and VY_REF than those of the whole group (Figure 2C and Table 1).

## Discussion

The aim was to determine whether wearing of compression garments results in high variability between individuals’ responses among a group of young, healthy, active subjects. The cluster analysis identified further distinct subgroups within the whole group of participants in each postural task, thus highlighting a large variation of individuals’ responses to the wearing of compression garments. In both postural tasks, only individuals who exhibited the highest values of linear center of pressure parameters in the REF condition (i.e., the less efficient or the more exploratory balance control at baseline) benefited from the wearing of compression garments to improve their balance control.

When statistics were performed on the whole group, our results showed that the wearing of compression garments did not significantly impact balance control. Previous studies about the effects of garments that interact with skin mechanoreceptors, such as compression garments, ankle braces, and taping, have also produced concordant findings with young, healthy, non-injured subjects and recreational sports people (Papadopoulos et al., 2007; Hijmans et al., 2009; Cavanaugh et al., 2016; Jaakkola et al., 2017; Willeford et al., 2018; Inglés et al., 2019; Medeiros Barbosa et al., 2019). Nevertheless, the clustering approach identified distinct subgroups of participants who responded differently to the wearing of compression garments. This result is consistent with some studies about the influence of external devices that stimulate skin mechanoreceptors on balance control, which have reported a high inter-individual variability in responses to cutaneous stimulation within a homogeneous group of young, healthy participants (Rogers et al., 2001; You et al., 2004; Pavailler et al., 2016). In both the stable and the unstable postural tasks, the clusters were differentiated according to the participants’ balance behavior at baseline and their ability to benefit—or not—from CG wearing to improve balance control. The participants who exhibited an efficient balance control at baseline, with low linear metrics of postural sway and/or high values of sample entropy at baseline (participants from cluster 1 in the stable task and from clusters 2 and 3 in the unstable postural task), did not take advantage from compression garments to improve their balance control. Only the participants who exhibited higher linear metrics of postural sway at baseline (participants from clusters 2 and 1 in the stable and the unstable postural task, respectively) benefited from the wearing of compression garments (illustrated by negative values of relative differences of linear center of pressure parameters between COMP and REF condition) to improve their balance control. Rogers et al. (2001) reported concordant findings while showing that the application of tactile stimuli benefited, to a larger extent, the subjects who swayed more during normal standing. These authors hypothesized that the subjects who swayed the most at baseline might present a moderate sensorimotor deficit, thus explaining why the addition of tactile stimuli led to a greater reduction in sway. Woo et al. (2017) formulated a similar hypothesis while postulating that the subjects with healthy postural control systems may not benefit from garments that interact with cutaneous receptors to enhance balance control. Further studies about the influence of external devices that stimulate cutaneous receptors (e.g., CG, ankle brace, and elastic bandages) on leg proprioception reported concordant findings when showing that the magnitude of the beneficial effects of cutaneous stimulation was inversely related to the participant’s proprioceptive acuity at baseline (Perlau et al., 1995; You et al., 2004; Cameron et al., 2008). You et al. (2004) also showed that external skin stimulation improved balance control only in subjects with low proprioceptive acuity. In the present study, we could therefore logically assert that the subjects who swayed the most benefited from the wearing of compression garments to improve their balance control because of a poorer balance control at baseline due to poorer lower limb proprioception.

Nevertheless, Pavailler et al. (2016) suggested that the ability to benefit from cutaneous stimulation provided by external supports was not only related to proprioceptive acuity but also to differences in how individuals weigh somatosensory cues for controlling their balance (i.e., differences in the preferred sensorimotor strategy). According to these authors, cutaneous stimulation would be of greater benefit to the subjects who rely more on gravito-inertial cues (gravity-dependent subjects) than on proprioceptive cues (support-dependent subjects). It is known that gravity-dependent subjects adopt an exploratory postural behavior while exhibiting larger postural sway to gain information from graviceptive signals (e.g., vestibular cues) more efficiently (Isableu and Vuillerme, 2006; Pavailler et al., 2016). Even though the subjects in the present study were asked to sway as little as possible in the context of a quiet stance paradigm, those who exhibited a greater postural sway could have a more exploratory postural behavior associated with a gravity-dependent sensorimotor strategy. However, exploratory movements have causal effects on the accuracy of affordance judgments (Stoffregen, 2003; Yu et al., 2011). Within the ecological approach to perception and action, the contents of perception are affordances (Gibson, 1966; Stoffregen, 2003). Affordances are neither properties of a subject nor properties of their environment, but they are emergent properties of the subject–environment system (Stoffregen, 2003). Compression garments act as a mechanically supportive framework which has a uniting function by nesting constricted mechanoreceptors, thus offering the opportunity to facilitate the pickup of information about sway-related movements in order to comply with the steadiness instruction. Compression garments did not enhance somatosensory perception *per se*, and one should rather consider a subject wearing compression garments as a system that provides an affordance for reducing postural sway. Stoffregen (2003) stated that affordances are “what one can do, not what one must do,” which means that affordances are opportunities that are not necessarily exploited by subjects (Riley et al., 1999). Actually, the exploitation of affordances is intimately linked to the availability of body movements (Stoffregen, 2003). An affordance can be viewed as a hidden property of a system that would become meaningful when the system is subjected to movement. Yu et al. (2011) showed, for example, that a certain amount of body sway was necessary for the perception of affordances. In the present study, the accuracy of affordance perception might have been higher in the participants who swayed more at baseline, those who have a typical profile of support-dependent subjects. With a greater sway, greater interactions certainly occurred between the cutaneous mechanoreceptors located under the compression garments, thus facilitating the pickup of information about sway-related movements. This could explain why only these subjects were able to take advantage from compression garments to reduce their postural sway.

This study showed that, among a sample of young, healthy, active subjects, only the participants who exhibited the higher amount of postural sway could benefit from the wearing of compression garments to comply with the steadiness instruction. Hence, it can be suggested that the ability to take advantage from the wearing of compression garments to improve balance control depends on the subjects’ balance/proprioceptive abilities at baseline or preferred sensorimotor strategy. Further experiments are needed to explore how preferred sensorimotor strategy and balance/proprioceptive abilities influence the ability to take advantage of compression garments to improve balance control. From a practical point of view, knowing that poor balance control is a predictor of sports injury risk (Hrysomallis, 2011), subjects at higher risk of injury are likely to benefit from the wearing of compression garments. Hence, wearing compression garments during sports practice could be viewed as a cost-effective injury prevention strategy that would have a specific impact on individuals at risk without having any deleterious effects on other individuals.

## Data Availability Statement

The raw data supporting the conclusions of this article will be made available by the authors, without undue reservation.

## Ethics Statement

The studies involving human participants were reviewed and approved by University of Pau and Adour Countries Ethics Committee. The patients/participants provided their written informed consent to participate in this study.

## Author Contributions

KB, FN, and TP designed the study. KB acquired the data. KB, FN, and NB analyzed the data. KB and FN wrote the manuscript. TP and NB revised the manuscript. All authors signed the final approval for publication.

## Conflict of Interest

The authors declare that the research was conducted in the absence of any commercial or financial relationships that could be construed as a potential conflict of interest.
